# Posture as Index for Approach-Avoidance Behavior

**DOI:** 10.1371/journal.pone.0031291

**Published:** 2012-02-15

**Authors:** Anita Eerland, Tulio M. Guadalupe, Ingmar H. A. Franken, Rolf A. Zwaan

**Affiliations:** 1 Department of Psychology, Erasmus University Rotterdam, Rotterdam, The Netherlands; 2 Max Planck Institute for Psycholinguistics, Nijmegen, The Netherlands; Linkoping University, United States of America

## Abstract

Approach and avoidance are two behavioral responses that make people tend to approach positive and avoid negative situations. This study examines whether postural behavior is influenced by the affective state of pictures. While standing on the Wii™ Balance Board, participants viewed pleasant, neutral, and unpleasant pictures (passively viewing phase). Then they had to move their body to the left or the right (lateral movement phase) to make the next picture appear. We recorded movements in the anterior-posterior direction to examine approach and avoidant behavior. During passively viewing, people approached pleasant pictures. They avoided unpleasant ones while they made a lateral movement. These findings provide support for the idea that we tend to approach positive and avoid negative situations.

## Introduction

Approach and avoidance are behavioral responses that are closely linked with emotion. According to an influential theory, emotions are tendencies to execute expressive behavior, like approach and avoidance, needed to achieve personal goals [1]. These behavioral responses are considered to be vital for survival [2]. On the one hand, this entails that we tend to approach pleasurable opportunities in order to promote our well-being and survival. On the other hand, we tend to avoid painful experiences in order to protect ourselves from harm.

Various studies provide support for the idea that the evaluation of a stimulus is linked to the tendency to move toward (positive evaluation) or away (negative evaluation) from the stimulus (see [3] for an overview). In a classic experiment, cards displaying stimulus words were presented to participants by a display box mounted on a response lever [4]. Half of the participants were instructed to pull the lever toward them whenever they liked the object displayed on the card and to push the lever away from them if they did not. The other participants received the reversed instruction. Results show that participants were faster when they had to push the lever away in reaction to stimuli they disliked and when they had to pull the lever in reaction to stimuli they liked. This is also true when participants are not explicitly asked to evaluate stimuli [5]. These results can be explained by the idea that negative stimuli activate avoidance behavior whereas positive stimuli activate more approach behavior [6].

Typically, most studies on approach and avoidance behavior examined arm movements as reaction to affective stimuli. As in the aforementioned experiments, pushing a lever away (i.e., arm extension) is associated with avoidance, whereas pulling the lever (i.e., arm flexion) is considered to be approach behavior. However, arm extension can also be associated with approach when people reach for a desirable object. Likewise, arm flexion can be associated with avoidance when people withdraw from an aversive stimulus. Therefore, these arm movements are ambiguous [7]. The ambiguity of arm flexion and arm extension regarding approach and avoidance behavior led to the hypothesis that not the movement itself but the effect of the movement is crucial. In other words, whether an arm movement is associated with approach or avoidance depends on whether the movement decreases (i.e., approach) or increases (i.e., avoidance) the distance between the stimulus and the self.

Given that arm movements are ambiguous regarding approach and avoidance, a more direct measure of these behaviors should be considered, like posture. In one study, the impact of the mere appraisal of the affective state of stimuli on posture was investigated. People viewed 20 pleasant, 20 neutral, and 20 unpleasant pictures for 6 s each while movements in their center of pressure were measured [8]. Whenever females (there were no effects for the male participants) viewed unpleasant pictures they shifted their balance backwards, which supports the notion of a relationship between motivated affective reactions and avoidance behavior. However, the researchers found no approach behavior in reaction to pleasant pictures and no main effect of picture valence on balance. This might be due to the fact that, given the extended exposure times, valence effects could be confounded by other factors (e.g., picture complexity). It is also possible that effects of valence on balance could not be detected due to the analysis that was used. With the analysis Hillman and colleagues [8] employed, the actual movement in reaction to a stimulus could not be examined.

To optimally assess the effect of valence on posture some points should be taken into account. First of all, evaluation of stimuli is immediate and without attention or awareness [9]. Effects of valence on posture are therefore expected to occur immediately after the presentation of a stimulus. Moreover, it is more informative to examine the trajectory of bodily movement rather than to reduce bodily movement to a single averaged position of the body in time. Finally, pictures belonging to different categories should be presented in random order. By doing so, changes in body balance are a more direct measure of the valence effect of a specific picture.

Here, we tried to address these issues and examine whether emotional pictures influence posture (1) when people passively view these pictures and (2) when people make a lateral movement. We included the lateral movement task because we were interested in postural changes over time. The participants had to move sideways to make the next picture visible, which allowed us to examine forward or backward deflection of this lateral movement as a function of picture content. Without such an explicit task, participants would have to stand still for a couple of seconds, which would have been difficult to motivate and might have led to random behavior. Thus, we examined whether (1) stance and (2) lateral movement are influenced by the pleasurableness of a picture. Given that we tend to approach pleasant stimuli and avoid unpleasant ones, we hypothesized that people lean slightly to the front in reaction to pleasant pictures and slightly to the back in reaction to unpleasant pictures.

## Methods

### Participants

Twenty undergraduate students (15 women) participated in this experiment. Their aged ranged from 18 to 30 with a mean of 22.3 years (*SD* = 3.3). In return for their participation the students received course credits. Participants provided informed consent by signing up online for this study.

### Apparatus

We used the Wii™ Balance Board (WBB) to measure participants' center of pressure (COP, a measure for body posture and balance). The COP measures produced by the WBB are as reliable and valid as those produced by an expensive laboratory-grade force platform [10]. Custom software was developed that enabled us to record event-related changes in COP and thus changes in body shift. The size of this shift is calculated from the change in weight distribution over the four (two left and two right) sensors of the WBB and is expressed in centimeters. Data were sampled at a rate of 33 Hz.

### Measures and procedure

Participants viewed 60 pictures from the International Affective Picture System (IAPS) [11] on a 15″ computer screen while standing on the WBB, placed 1 m. away. Twenty of these pictures were pleasant (e.g., scenes of erotica, families and animals), twenty were neutral (e.g., neutral faces and scenes of household objects), and twenty pictures were unpleasant (e.g., sad and scared people, see Table 1). We used the following pictures from the IAPS: pleasant – 1463, 2030, 2071, 2655, 4150, 4255, 4520, 4533, 4542, 4601, 4610, 5814, 5830, 7260, 7350, 7430, 7470, 7508, 7580, 8461; neutral – 2190, 2440, 2480, 2840, 5130, 7000, 7004, 7006, 7035, 7040, 7041, 7090, 7150, 7175, 7217, 7490, 7491, 7705, 7950, 9360; unpleasant – 2141, 2205, 2276, 2700, 3181, 3300, 6561, 6562, 7361, 9007, 9041, 9180, 9320, 9415, 9417, 9432, 9435, 9470, 9561, 9830. Before the experiment started, the WBB was calibrated for each participant to make sure that the neutral body posture was consistent with the center of a fixation cross. Participants made a new picture appear by keeping their COP within a certain circle in the middle of crosshairs displayed on the screen. After a picture was presented for 1 s (i.e., passively viewing phase) a white arrow appeared in the middle of the picture. This arrow always pointed left or right. Participants were instructed to lean towards the side directed by the arrow (i.e., responding phase). When the COP crossed a threshold, the picture disappeared and participants again saw the crosshairs displayed on the screen. The next picture appeared whenever they kept their COP within a circle in the middle of the crosshairs. We recorded the medio-lateral and antero-posterior excursions of the COP. Data were analyzed separately for both phases of the experiment. The order in which the pictures appeared was randomized and the direction of the arrows was counterbalanced within and across participants. This study was approved by our Institutional Review Board.

**Table 1:** Mean valence and arousal rates (+*SD*) by kind of IAPS pictures used in this experiment.

	IAPS rating		Participants'rating	
	Valence	Arousal	Valence	Arousal
Pleasant (*n* = 20)	6.99	4.87	6.19 (1.95)	3.25 (2.24)
Neutral (*n* = 20)	4.84	2.45	4.18 (1.63)	1.34 (0.79)
Unpleasant (*n* = 20)	2.75	4.88	2.86 (1.75)	4.28 (2.46)

## Results

All trials with very fast (<3 SD) or very slow (>3 SD) response times were excluded from analysis. These were defined as the time elapsed between the arrow's onset and the response's apex. As a result, only trials with a response time between 620 and 2480 milliseconds remained. This procedure led to the removal of 4.6% of the data.

**Figure 1:**
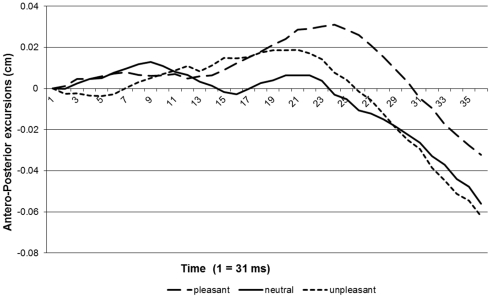
Baseline corrected averaged response curves of y-axis movements during stance.

**Figure 2:**
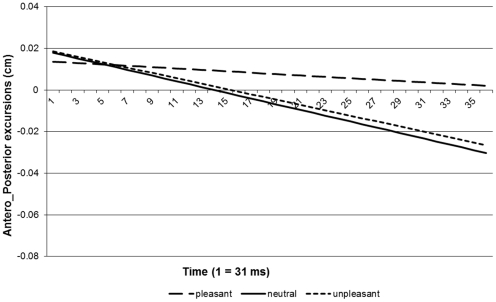
Linear models based on the baseline corrected averaged response curves of y-axis movements during stance.

The data were baseline-corrected, so that all trials commenced from the same (0.0) coordinate (see Fig. 1). We divided the data into two sections for analysis: the first phase, passive viewing of the pictures, and the second phase, lateral movement. Average responses were then calculated for each subject, condition and phase. We were interested in whether people leaned more forward while viewing a pleasant picture as compared to a neutral one and in whether people leaned more backward while viewing an unpleasant picture as compared to a neutral one (passive phase). Therefore, we fitted a linear model on the responses with subject as a random effect (Fig. 2). Adding a linear effect of condition improved the fit or our model significantly (Δ -2* log-likelihood = 5.54, *df* = 1, *p*<.05). The slope of the pleasant condition differed significantly from that of the neutral condition. However, the slope of the unpleasant condition did not (see Table 2). These results indicate that people show an approach but no avoidance response while they passively view emotional pictures.

**Table 2:** Coefficient values for a linear model (passively viewing phase).

	Coefficient	SE	*t*-value	*p*-value
Neutral *(reference condition)*	0.002	0.001	2.149	0.032
Pleasant	0.001	0.000	2.126	0.034
Unpleasant	9.11^E^ -0.5	0.000	0.185	0.853

**Figure 3:**
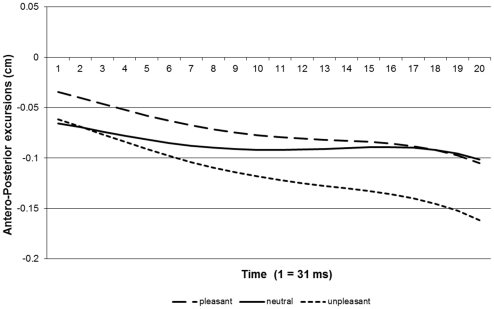
Averaged response curves of y-axis movements during lateral movement.

**Figure 4:**
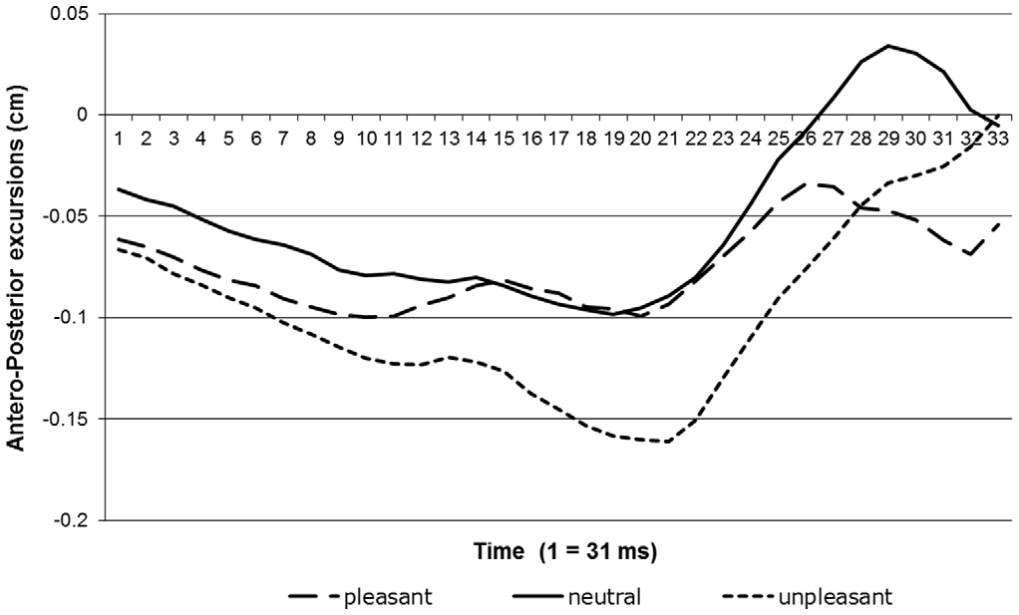
Quartic models based on the averaged response curves of y-axis movements during lateral movement.

Figure 3 shows the results of the lateral movement phase. The responses' starting points are equal to the ending point of the responses in the first part of the trials. Because the responding phase was not time-limited, as was the passively viewing phase, response times differed over trials. We constrained the analysis to data within the first 620 ms (20 data points). We did so because at that point participants started to reach their maximum amplitude of responding (see Fig. 3). This means that from that point on participants started to reestablish their balance and changes in COP are a result of this process rather than participants' response to the arrow. Given the nature of the movements in this second phase (see Fig. 3), we fitted a quartic model to our data. Again, we compared the linear effects (see Fig. 4) for the pleasant and unpleasant responses with the neutral response. We found that adding a linear effect of condition improved the fit or our model significantly (Δ -2* log-likelihood = 10.08, *df* = 1, *p*<.05). Subjects tend to lean backward while shifting their balance to the left or to the right. However, they lean more backward in response to unpleasant pictures than to neutral ones. There was no significant difference in backward motion between the pleasant and neutral conditions (see Table 3).

**Table 3:** Coefficient values for a quartic model (responding phase).

	Coefficient	SE	*t*-value	*p*-value
Neutral *(reference condition)*	0.087	0.053	16.881	8.55^E^ -57
Pleasant	−0.047	0.028	−1.712	0.087
Unpleasant	−0.087	0.028	−3.162	0.002

## Discussion

The purpose of this study was to investigate postural changes, instead of arm movements, as an index of people's tendency to approach pleasant pictures and to avoid unpleasant ones (1) while passively viewing emotional pictures and (2) while making a lateral movement. We hypothesized that by using a sensitive procedure, we would be able to detect that people lean slightly to the front in reaction to pleasant pictures and slightly to the back in reaction to unpleasant pictures as compared to a response to neutral pictures and this is what we found. In the first phase of picture viewing, before the sideward movement was initiated (passive phase), people tended to approach pleasant pictures, but we did not find an avoidance movement in reaction to unpleasant pictures. When people made a lateral movement they leaned backward independent from the kind of picture they viewed. However, people leaned significantly more backward in response to unpleasant pictures. It might be that leaning backward is a biomechanical necessity when moving sideways.

Although approach and avoidance responses are thought to be immediate and automatic [2], we only found an immediate approach effect and a delayed avoidance effect. These results are consistent with previous studies on this topic. In the study by Hillman et al. [8] a backward movement was found in females in reaction to unpleasant pictures after two seconds. No approach behavior was found in reaction to pleasant pictures during the six-second duration of each trial. Whether there was an immediate approach response could not be investigated. In another study, body sway was investigated in reaction to happy, neutral, and angry faces. Three seconds after stimulus onset there was no difference in body sway between neutral and happy faces [12]. More recently, delayed avoidance reactions were found in reaction to facial expressions [13]. All these results indicate a relatively slow effect of negative stimuli on posture.

The fact that we found an immediate approach and a delayed avoidance effect might be explained by the nature of the pictures that we used. Half of the unpleasant pictures used in this experiment depicted scenes with sad and scared people. To be able to respond to these scenes it is necessary to understand what the scene is about. Perhaps understanding the scenes of the unpleasant pictures required more time than understanding the pleasant pictures, which included mostly food and attractive and happy people.

The present study shows that the methodology can be used in the studies addressing the behavioral aspects of emotion. As several theories of emotion assume that emotions are associated with an automatic approach or avoidance tendency, the present methodology can be helpful in addressing these assumptions. Also in the field of clinical psychology the present methodology could be used. For example, several theories assume an automatic avoidance tendency for fearful stimuli in anxiety disorders and an automatic approach to substance related stimuli in addictive behaviors. Currently, these behavioral biases are usually investigated using arm-movements (as a proxy for approach and avoidance behavior). The present study shows that posture might be a valid alternative for these proxy measures.

In sum, this study examined postural responses in reaction to emotional pictures. Our findings provide support for the idea of balance as indicator of an approach mechanism for positive information as well as an avoidance mechanism for negative information.
