# Correction: Posture as Index for Approach-Avoidance Behavior

**DOI:** 10.1371/annotation/3ea5cb26-e73e-4836-887d-d6318281e0f2

**Published:** 2012-05-24

**Authors:** Anita Eerland, Tulio M. Guadalupe, Ingmar H. A. Franken, Rolf A. Zwaan

The images for Figures 3 and 4 were incorrectly switched. The image that appears as Figure 3 should be Figure 4, and the image that appears as Figure 4 should be Figure 3. The figure legends appear in the correct order.

